# Effect of different fillers on thermal conductivity, tribological properties of Polyamide 6

**DOI:** 10.1038/s41598-023-27740-y

**Published:** 2023-01-16

**Authors:** Gyorgy Czel, Anna Sycheva, Dora Janovszky

**Affiliations:** 1 Institute of Ceramic and Polymer Engineering, University of Miskolc, Miskolc-Egyetemvaros, 3515 Hungary; 2 ELKH-ME Materials Science Research Group, University of Miskolc, Miskolc-Egyetemvaros, 3515 Hungary

**Keywords:** Engineering, Materials science

## Abstract

An influence of different filler types and filler content on the thermal and abrasive wear properties of polyamide-6 is investigated. Al_2_O_3_, MgO, two glass powders with different SiO_2_ contents, and natural zeolite powder were selected as fillers. The fillers individually were added to the polymer matrix in proportions of 50 and 70% by weight. A hybrid filler-containing composite was created by mixing PA6/70 wt% MgO and PA6/80 wt% zeolite. The results show that the thermal conductive enhancement factor is highest for PA6/70 wt% Al_2_O_3_ (145%) and PA6/hybrid fillers 75 wt% (92%). The Lewis-Nielsen and Reciprocity models agreed with the measured data with less than 26% deviation, except for the MgO-loaded composites. In the case of a hybrid composite, the additive model proves to be a good approximation. The abrasive effect of the different fillers was characterised by the volume loss of the steel pin using the pin-on-disc method. A new parameter is developed that considers the thermal conductivity enhancing effect of the fillers and their abrasive effect. In addition to ceramic fillers, aluminium-hydro-silicate, e.g. natural zeolite, and their mixtures offer new opportunities for the development of thermally conductive composites, as they are more economical to use in manufacturing processes.

## Introduction

In recent decades, we have been living in the age of 5G technology, miniaturisation, and the fast development of power density. The global electric vehicle fleet expanded significantly over the last decade (electric cars, bicycles), and this requires the rapid development of electrical equipment, as sizes are reduced, but performance needs to be increased. Therefore heat dissipation is becoming one of the most critical technological challenges^1–3^. Accumulated heat can cause severe damage and significantly reduce a device’s lifetime. Plastics are increasingly displacing metals due to their superior properties, such as lightweight, tailorable thermal conductivity, low-cost processing, and excellent resistance to corrosive conditions when compared with metals. Energy is saved through low-temperature processing. Plastics have poor thermal conductivity, usually in the range of 0.1–0.5 W/mK^4^, but the appearance of plastic composites with good thermal conductivity can solve this problem^5^. Thermally conductive fillers, carbon fibers^6–9^ or metallic fillers such as copper^10^, silver^11^, and aluminium^12^ are most often used to increase thermal conductivity in polymers. There is also a growing demand for suitable good, thermally conductive plastics that are electrically insulating. In this case, aluminium oxide (Al_2_O_3_)^13,14^, aluminium nitride (AlN)^15^, boron nitride (BN)^16–19^ boron carbide (B_4_C)^20^, and silicon carbide^21^ are usually introduced into polymers by solution mixing or melt processing. An increase in thermal conductivity can be enhanced not only by the use of a single ceramic filler, and thus, hybrid fillers are commonly introduced in polymer composites^22,23^.

The heat conduction process of thermally conductive polymer composites becomes more complex owing to added filler, including thermal conduction pathways, thermally conductive percolation, and thermoelastic coefficient mechanism^24^. Besides to the good thermal conductivity of the filler, volume fraction and dispersion are of much greater importance^25^. Most fillers are isolated if there is a reduced good thermal conductivity filler content in the polymer composite. Existence of the polymer phase results in a high thermal resistance at the interfaces between fillers and the polymer matrix. In order to have good thermal conductivity, there must be enough filler to create continuous thermal conduction pathways or networks in the polymer composite. Thermally conductive but electrical insulator plastics have a much more uniform temperature distribution than unfilled ones. Increasing the amount of filler, of course, increases the thermal conductivity, but it can impair other physical characteristics. Due to the ceramic filler, the amount of tool wear during production appears, and injection moulding becomes more and more difficult. Hybrid fillers in polymers offer new possibilities for increasing thermal conductivity. Combining two different particle sizes^24^ or two different types of fillers^25^ provides a higher thermal conductivity than any one in the combination.

Polyamide 6 (PA6) is used in large quantities in injection moulding workshops, is chemically compatible with other materials, has good mechanical properties, heat resistance, electrical insulation, and also easy processing performance, and therefore is suitable for use as a thermally conductive composite matrix. Boron nitride is most often added to the electrically insulating polyamide matrix to increase thermal conductivity^16–18^, but the thermal conductivity of BN depends on the orientation direction. There is still little literature on isotropic, thermally conductive, but electrically insulating polyamide materials. Isotropic, electrically insulating but thermally conductive polyamide composites can be formed using oxides (Al_2_O_3_, SiO_2_, MgO) and silicate filler. The thermal conductivity of silicon dioxide varies from 0.33 to 1.7 W/mK. SiO_2_ is an excellent insulator (~ 10^15^ Ωm), and the thermal expansion coefficient is very low (5.6 × 10^–7^ 1/K). PA6 with short glass fibers composites are widely used for various automotive applications^26^. Ranganathan et al.^27^ used glass fiber and beads as filler for the PA6 matrix, and they reached 0.6 W/mK thermal conductivity when the glass bead content was 30 wt.%. The synthetically produced Al_2_O_3_ has moderate thermal conductivity (20–40 W/mK) but excellent electrical insulation (1 × 10^12^–1 × 10^13^ Ωm). This chemically stable material has very high strength and hardness at low density. Ren et al.^28^ reached 1.18 W/mK when the content of modified Al_2_O_3_ was 70 wt.% in the PA6 matrix. Y. Kim et al.^29^ used 50–70 wt% Al_2_O_3_ filler, and the thermal conductivity was increased from 0.25 to 1.16 W/mK. Al_2_O_3_ is a promising filler in future industrial applications^14^ Wang et al.^30^ prepared PA6 composites containing AlN and BN, each of which 50 wt.%, and the thermal conductivity were 0.799 and 0.93 W/mK, respectively. The synthetically produced MgO also has high thermal conductivity (30–60 W/mK) and good electrical insulation (1 × 10^12^–1 × 10^13^ Ωm at room temperature). However, MgO can react with moisture and is very prone to agglomeration. Zhang et al.^31^ fabricated a PA6 composite with a MgO nanoparticles-decorated carbon fiber hybrid filler. They reached 0.748 W/m·K at 20 wt % hybrid fillers. Wang et al.^32^ organic montmorillonite (> 30%) filled PA6 foams were prepared. They found that the thermal conductivity of PA-OMMT composite foams increased until 60 wt% of montmorillonite content.

Herein, we investigated several types of filler in terms of thermal conductivity, mechanical properties, and abrasion to obtain the most favourable electrical insulation PA composites. In this work, magnesium oxide, alumina, two glasses with different SiO_2_ content powders, and natural zeolite were used to improve the properties of the polymer matrix. Among the composite manufacturing methods, injection moulding has lower costs, better time efficiency, and improved processability. In addition to thermal conductivity, tool wear was measured by a pin-on-disc wear test, where the wear of a steel ball used as a pin was investigated.

## Materials and methods

### Materials

Commercially available PA6 (DOMAMID PW 6FC NC) supplied by DOMO Chemicals GmbH Germany was used as matrix resin in this work. This material is used in large quantities in the composite industry.

The natural zeolite, crystalline aluminium-hydro-silicates, was commercially available from Geoproduct Ltd. and originated from Mád (Tokaj region, Hungary). The mineral composition is presented in Table 1. This natural zeolite powder also contains smectites which are clay minerals.

**Table 1:** Mineral composition of the natural zeolite used in the experiments (on a dry basis).

Phase name	wt.% Rietveld	Empirical formula
Smectite group	45.8	M_0.33_Al_2_(Si_3.67_Al_0.33_)O_10_(OH)_2_^a^
Clinoptilolite	33.5	Ca_1.9_Na_1.76_K_1.05_Mg_0.17_Al_6.72_Si_29.2_O_72_·23.7(H_2_O)
Cristobalite	8.8	SiO_2_
Sanidine	2.6	K_0.75_Na_0.25_AlSi_3_O_8_
Quartz	2.3	SiO_2_
Amorphous	7.0	–

^a^M = Ca^2+^ or Mg^2+^

.

Magnesium oxide (MgO) powder was supplied by Lehmann and Voss and Co. (Germany). Alumina (Al_2_O_3_) powder was manufactured by Imerys Fused Minerals Villach GMBH (Austria). Two glass powders (hereinafter referred to as Fritt1 and Fritt2) were used as reinforcement fillers. These powders were obtained from Cerlux Ltd., Hungary. The composition of Fritt1 and Fritt2 is presented in Table 2. Fritt1 has got relatively high SiO_2_ and MgO content. Fritt2 ceramic powder has high B_2_O_3_ glass content. The properties of the PA6 resin and fillers are presented in Table 3. Polyamide composites containing 50, 70, and 80 wt.% fillers were tested. The surface of the fillers was not modified.

**Table 2:** Composition of Fritt1 and Fritt2 used in the experiments (on a dry basis).

Phase name	Fritt1 wt.%	Fritt2 wt.%
SiO_2_	57.23	46.86
CaO	9.89	7.63
ZnO	8.06	–
ZrO_2_	6.63	–
Al_2_O_3_	6.29	1.56
K_2_O	3.35	0.34
B_2_O_3_	3.29	28.03
MgO	3.07	0.76
Na_2_O	–	14.45
Other	2.19	0.37

**Table 3:** Properties of PA6 and different fillers.

Properties	PA6	Natural zeolite	MgO	Al_2_O_3_	Fritt1	Fritt2
Thermal conductivity, W/mK	0.19	1.2	30	36	1.24	1.21
Density, 10^3^ kg/m^y^	1.14	1.99	2.20	3.54	2.68	2.57
Purity, %			98.5	> 99		
The volume mean diameter, µm		78.2	232.3	25.1	15.1	16.6
Mohs’ hardness		4–5	5.8	9	4–5	4–5

The properties of the PA6 resin and fillers are presented in Table 3.

### Composites preparation

Before blending, PA6 was dried at 90 °C for 2 h in dry air drier to remove the moisture from the PA6. All the different fillers were dried in the drying furnace for a minimum of 48 h at 200 °C. A blade mixer was used for dry powder mixing. PA6 matrix-based composites manufactured by Thermo-Haake PolyLab System with a Rheomix 610p laboratory inner mixer (Thermo Fisher Scientific Inc.) or a LabTech twin-screw compounding extruder (LabTech Engineering Company Ltd.) were used. The screw speed was 290 1/min, the feeding speed of 19 1/min and the melting temperature of 250–280 °C. The diameter of the screw in the extruder machine was 20 mm, and the length/diameter ratio was 40. The fixed weight fractions of filler were 50, 70 wt.%. The number after the name of the filler indicates the weight percentage (PA6/MgO-50 means composition with 50 wt% MgO filler). To increase thermal conductivity, hybrid filler was also formed from the different available powders. Two polyamide composites were mixed by a 50–50% weight ratio: one containing 70 wt.% of MgO and one containing 80 wt.% of natural zeolite (hereinafter referred to as PA/MgO-70/Zeo80-50/50). Finally, the mechanically granulated reinforced thermally conductive PA6 composites were obtained. After this, they were dried at 75 °C for 8 h while the moisture content of the material fell below 0.05%. The granular composites were injection moulded to obtain specimens with standard flat shapes by a KM80-160C1 injection moulding machine (KraussMaffei Technologies GmbH. Germany).

### Characterisation

The thermal conductivity of the composites was measured at room temperature by a C-Therm TCI (C-Therm Technologies Ltd. Canada) apparatus. The testing samples were 20 mm in diameter and 2 mm thick.

The Shore D hardness measurements were measured by Zwick/Roell equipment. Ten hardness measurements per piece were performed, and a minimum of four pieces were examined.

The thus prepared composites were cryogenically fractured in liquid nitrogen. A micrograph of fractured surfaces was acquired by a Hitachi S4800 scanning electron microscope (SEM, Hitachi Ltd, Japan) equipped with a BRUKER AXS type energy dispersive X-Ray spectrometer (EDS, Bruker GmbH, Germany) and a Helios G4 PFIM-SEM (Thermo Fisher Scientific Inc., USA).

Non-isothermal analyses were carried out using a Netzsch STA 449 F3 Jupiter TG-DSC differential scanning calorimeter. About 20 mg of each sample were weighed quite accurately in the aluminum DSC pan, placed in the DSC cell, and then, it was heated from 28 to 300 °C at a rate of 10 °C/min under a purified argon atmosphere. Each sample was kept for 3 min at this temperature. After that, they were cooled to 100 °C at the same scanning rate. Finally, the samples were reheated to 280 °C. All the endothermic measurements were taken from the second heating scan of the samples to remove the previous thermal history, and the exothermic measurements were taken from the first cooling scan. The degree of crystalline (X_c_) was calculated from melting enthalpy values using the following equation:

1 $${X}_{c} \left(\%\right) = \frac{{\Delta H}_{m}}{{\Delta H}_{m}^{0}}\cdot 100\%$$

where ΔH_m_ is the melting enthalpy of the samples, and ΔH^0^_m_ is the enthalpy value of melting of the 100% crystalline form of PA6 (240 J/g)^33,34^.

Wear tests of the PA with different fillers were performed under dry sliding conditions using a pin-on-disc tribometer (CSEM Instruments, Switzerland) with continuous rotation. The coefficient of friction was recorded during the tests. A peripheral speed of 0.6 mm/s speed was used and the sliding distances were 5000 and 7500 m. The pin was a steel ball (745 HV) 8 mm in diameter with a load of 10 N. The abrasive effect of the different fillers was characterized by the volume loss of the steel ball. The smaller diameter of the ellipse on the abraded surface of the ball was measured with an optical microscope. The volume of the missing spherical cap of the ball was calculated. The specific wear rate (W_r_) was defined according to:

2 $${W}_{r}=\frac{\Delta V}{S\cdot F} ,\frac{{\mathrm{mm}}^{3}}{\mathrm{N}\cdot \mathrm{m}}$$

where ΔV is the volumetric loss of the ball after sliding, S is the sliding distance, and F is the load. After the tests, the wear tracks were characterized with an optical and scanning electron microscope.

## Results and discussion

### Morphology and particle size distributions of fillers

**Figure 1:**
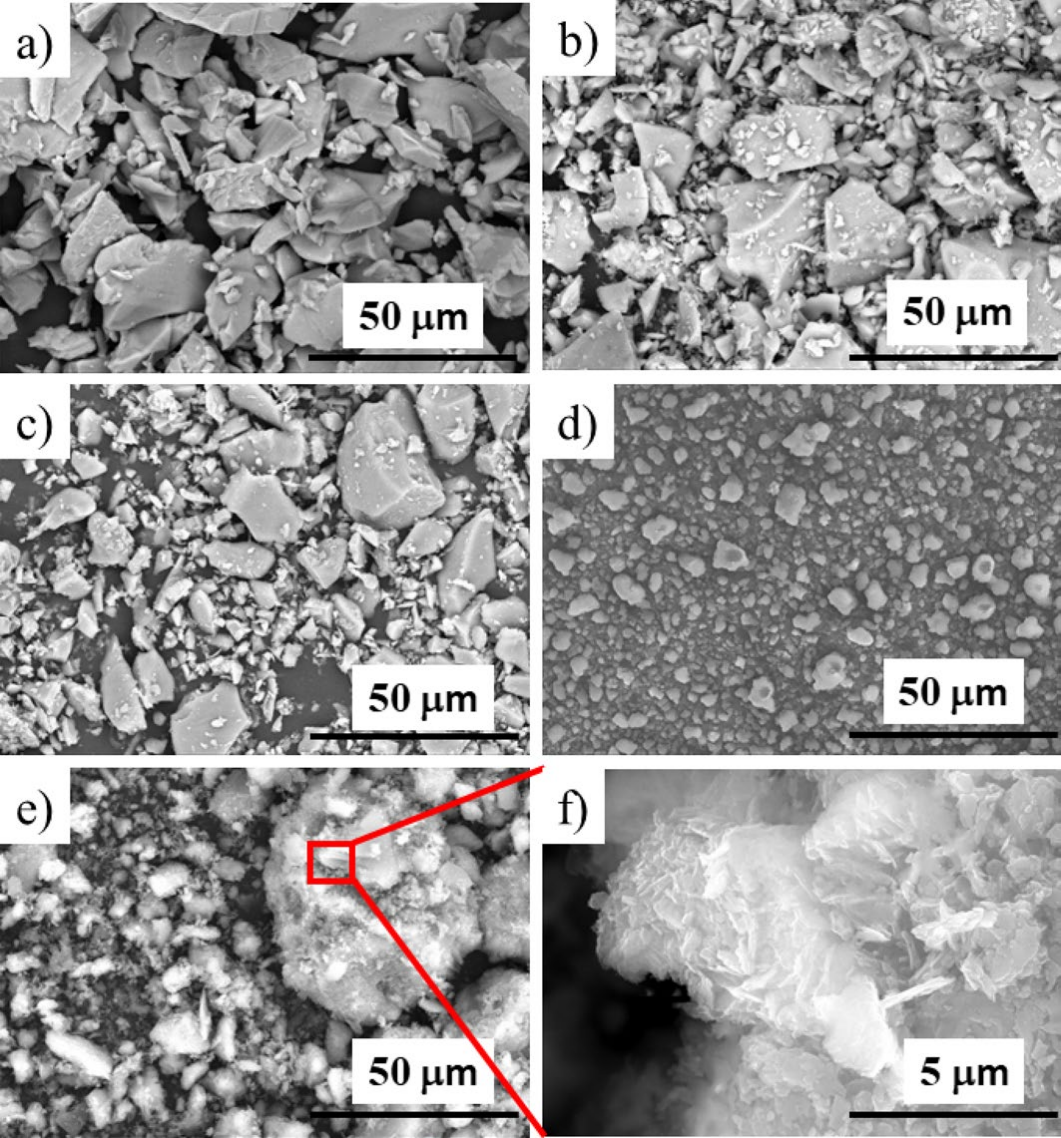
Scanning electron microscopic images of the different types of fillers: (**a**) Al_2_O_3_, (**b**) Fritt1, (**c**) Fritt2, (**d**) MgO, (**e**) and (**f**) natural zeolite.

**Figure 2:**
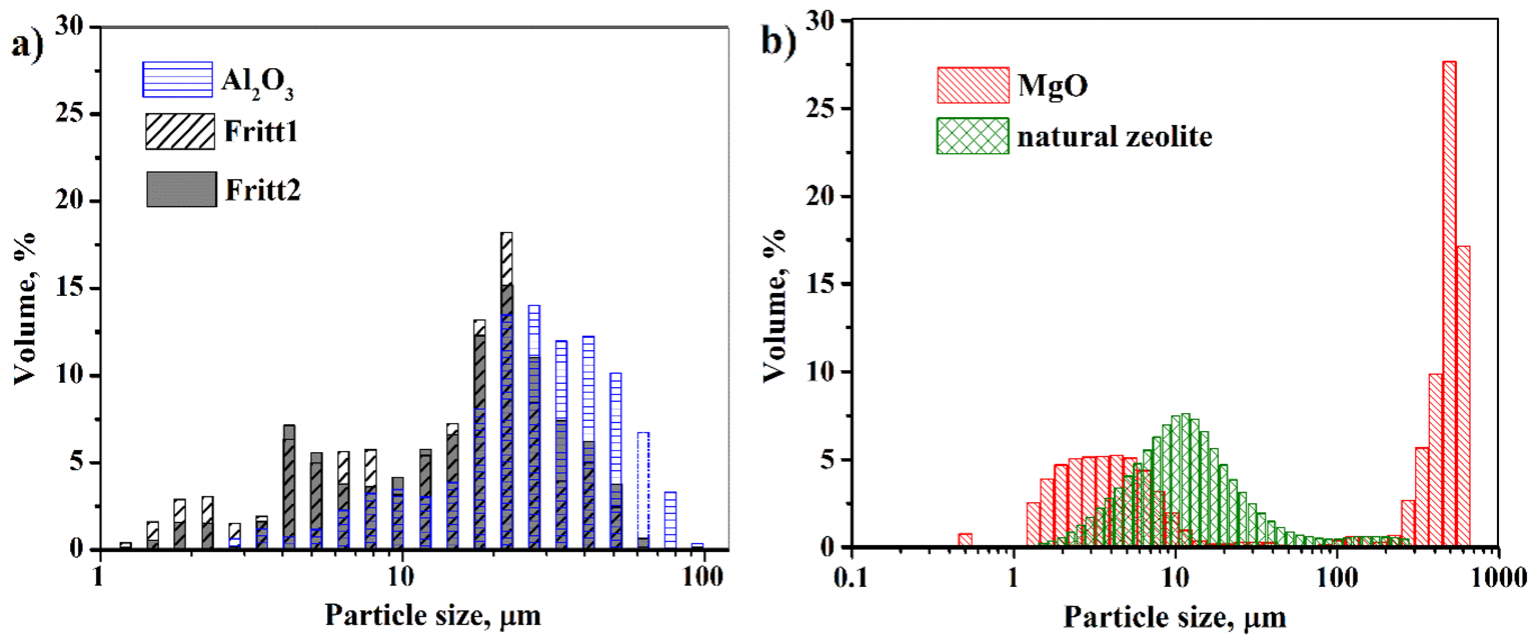
Particle size distribution of the different fillers.

The goal was to create an isotropic composite with good thermal conductivity and less tool wear, so the morphology of the fillers was examined first. As can be seen in Fig. 1a–c, Al_2_O_3_ and Fritt1-2 particles have an irregular shape with sharp fracture edges. For all three powders, based on the particle size distribution (Fig. 2a), the particle sizes are below 100 µm and the distributions show near-normal. The median (D_50_) cumulative particle size is 25.14 µm, 15.05 µm, and 16.63 µm in the case of Al_2_O_3_, Fritt1, and Fritt2, respectively. By contrast, natural zeolite, and especially MgO, form larger and nearly spherical aggregates (Fig. 1d,e). Apparently, the aggregates have no sharp edges. MgO powder aggregates very quickly. The particle size distribution confirms the presence of aggregates in the case of MgO powder (Fig. 2b). Half of the volume of MgO consists of particles below 10 µm, while the other half consists of particles of 300–500 µm. The aggregates for MgO and natural zeolite powders comprised 1–30 µm plate-shaped pieces with a thickness of less than 1 micron (Fig. 1f). Natural zeolite consists of 45 wt% smectite, which has a layered structure. These layers are stacked together by weak van der Waals forces to form a clay particle^35^. This structure allows nanocomposites to be created using a clay mineral filler^35^. The median cumulative particle size of MgO and natural zeolite is 33.90 µm and 28.56 µm, respectively.

### Microstructure of composites

**Figure 3:**
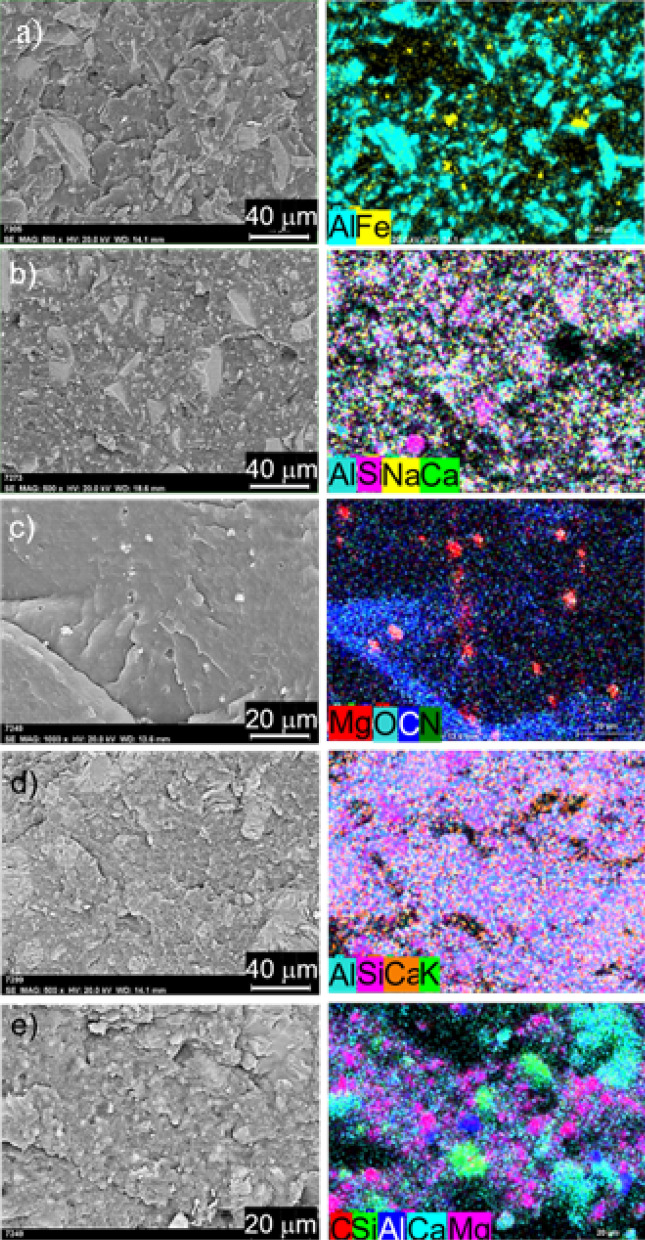
SEM images and the elemental mapping of composites containing 70 wt% of different types of filler composites in PA6 matrix: (**a**) Al_2_O_3_, (**b**) Fritt1, (**c**) MgO, (**d**) natural zeolite, and (**e**) PA/MgO-70/Zeo80-50/50 hybrid filler.

The cryo-fractured surfaces of composites with 70 wt.% filler content were investigated to determine the distribution state of fillers in the PA6 matrix. EDS elemental mappings of the composite cross-sections show the location of the filler particles in the matrix even more spectacularly. The zeolite, Al_2_O_3_, Fritt1, and Fritt2 filler particles exhibited relatively good dispersion into the PA6 phase (see Fig. 3a,b,d). In the case of 70 wt% filler content, the filler volume content of all PA6 composites is about 50% except Al_2_O_3,_ where 43%. With such a large amount of the second phase, it can be assumed that a thermal conduction pathway forms. A gap thinner than a micron is visible around some filler particles. The adhesion force was lower than the shrinkage force during the curing process. In all cases, the gap between the filler and the matrix impairs thermal conductivity. In the case of Al_2_O_3_, Fritt1, and Fritt2 filler particles, one can observe two characteristics of the filler/PA6 composites fracture surface: (I) few particles pull out from the matrix, and some holes appeare; (II) debonding of the filler particles/PA6 interface, the direction of crack propagation was deflected by the particle and further propagated at the particle/matrix interface. Natural zeolite is well dispersed in the PA6 matrix; in fact, it appears that the zeolite content is more than 57.22 V/V%. Zeolite particles are composed of micro-nano scale layers, as can be seen in Fig. 1e, which probably break down into individual particles to form micro-nano-sized filler. The amount of MgO is much less than expected in the cross-sections studied (Fig. 3c). This is probably because MgO is highly agglomerated, and its distribution is not homogeneous.

In the case of hybrid filler (PA6/Mg70-PA6Zeo80-50/50), smaller MgO particles fill the volume between the larger zeolite particles (see Fig. 3e) and form a higher packing density of the fillers in the matrix. It is worth noting that, in this case, the distribution of MgO particles is homogeneous; they may have disintegrated during the mixing of the two types of composites. Due to the higher volume content of the fillers particles, which is 62%, the distance and resistance among adjacent conductive fillers decreased, and the thermal conductivity can increase. Therefore, the formation of more effective conductive pathways or networks in the matrix has great importance for enhancing thermal conductivity.

### Hardness of composites

**Figure 4:**
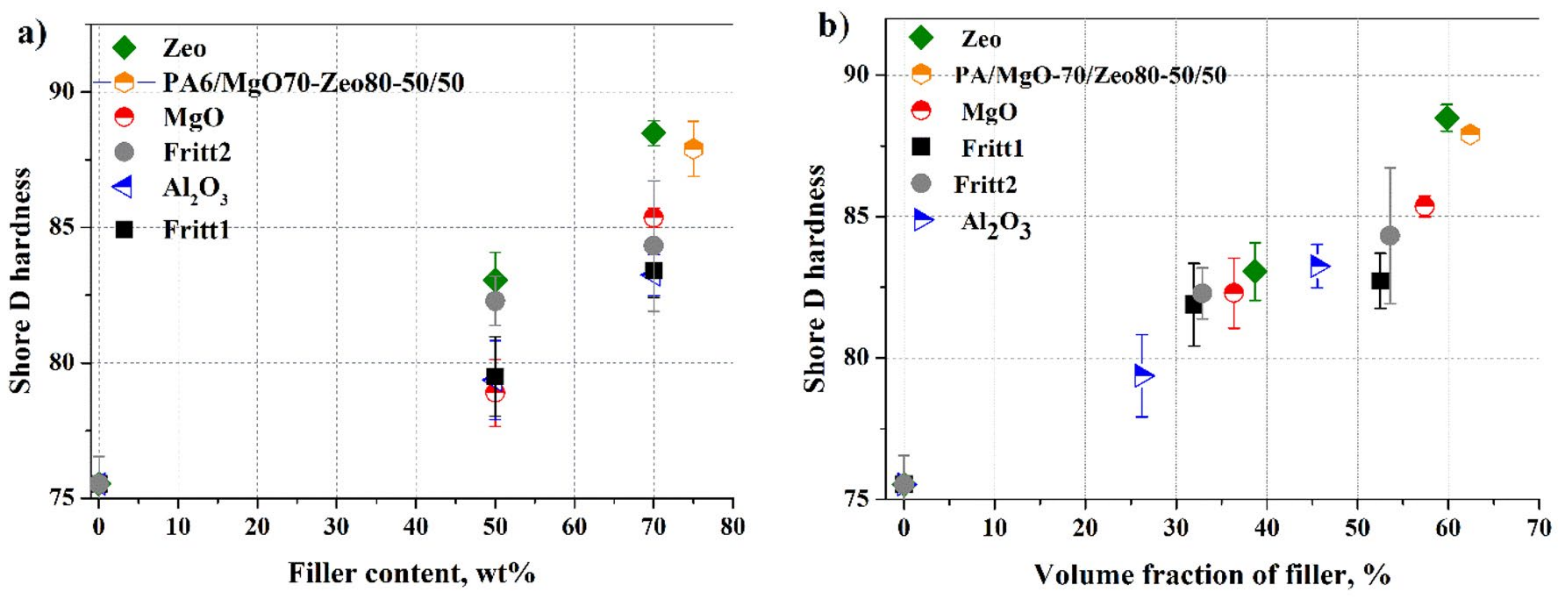
Dependence of Shore D hardness on weight (**a**) and volume percentage (**b**) for different types of filler in PA6 composites.

The mechanical property, such as surface hardness (Shore D) of PA6 composites as a function of volume concentration (which is calculated from the material density) is shown in Fig. 4. As can be seen, the surface hardness continuously increases with increasing filler content. In the case of 50 wt%, the composite containing Al_2_O_3_ has the lowest hardness (79 ShoreD), while the composite containing zeolite has the highest (83 ShoreD). In the case of 70 wt% filler content, also the Al_2_O_3_ composite has the lowest hardness, while the zeolite composite has the highest (88.5). This might be a surprising result, since Al_2_O_3_ filler has the highest hardness among the investigated fillers, and zeolite is almost half as hard. However, the difference in density between fillers must be taken into account. The density of the zeolite is lower than that of Al_2_O_3_, and because of this, the composite with zeolite content has a larger volume of filler at the same weight percentage (Fig. 4b) so that a higher hardness can be achieved than in the case of Al_2_O_3_ . This makes it possible to produce especially hard composite (88 ShoreD), even if a filler with a lower hardness than alumina’s hardness is used.

### Thermal properties

**Figure 5:**
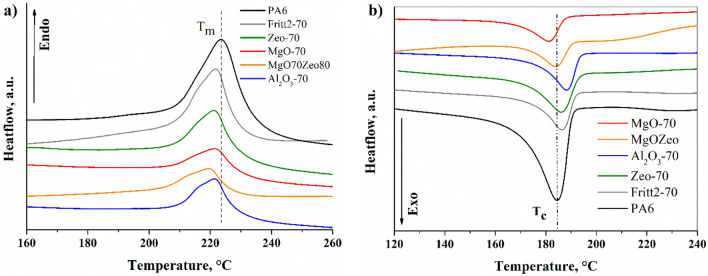
DSC heating (**a**) and cooling (**b**) curves showing the melting and crystallisation behaviours of the representative composite samples as indicated in the graphs.

The melting and nonisothermal crystallisation behaviours of the samples were comparatively investigated using DSC, and the results are presented in Fig. 5 and Table 4. Here, a second heating scan was considered to eliminate the previous thermal history of the composites. The onset crystallisation temperature (T_c-onset_), crystallisation peak temperature (T_c_), enthalpy of crystallisation (ΔH_c_), melting peak temperature (T_m_), enthalpy of melting (ΔH_m_), and degree of crystallinity (X_c_), are summarised in Table 4. The stable crystalline form of PA6 polymer is the monoclinic α-form crystal, which generally crystallises at T_c_ > 150°C^36^. The pseudo-hexagonal γ-form crystals can crystalise in the quiescent melt at T_c_ < 150^36,37^. In the case of the pure PA6 sample, a broad and high endothermic complex peak can be seen at T_m_ = 223.6 °C (Fig. 5a). During melting, two processes take place, supported by the shoulder in the lower temperature part of the peak (≈ 215 °C) and the first derivative of the process. The different types of fillers modified the melting process of PA6. The peak temperatures shifted towards lower temperatures for all fillers (Table 4), and the shoulders became more dominant (Fig. 5a). Based on these phenomena, two kinds of crystallites, either of the same crystal form but having two different crystal thickness or of a different kind, namely α- and γ-form crystals melting process takes place. It is known from literature that, the melting peak temperature of α-form (≈ 220 °C) of PA6 is higher than γ-form (≈ 212 °C)^38,39^. Al_2_O_3_^39^, SiO_2_^40^, glass^41^ and montmorillonite^38^ fillers promote γ-form crystallisation. Based on the literature data, this suggests that, in our case, too, γ-form crystals are present in the composites in addition to α-form. Increasing filler content from 50 to 70 wt% does not significantly change the melting peak temperature. However, the shoulder is becoming more pronounced, which indicates an increase in the amount of γ-form crystal. The maximum depression in T_m_ for initial PA6 (223.6 °C) is observed for Zeo-80 filler (213.4 °C). The enthalpy of melting and the crystalline volume fraction for each filler follow the same trend and decrease with increasing the filler content. The reduction of the crystalline fraction can be due to several reasons; on the one hand, the fillers increase the viscosity of the melt, and on the other hand, the fillers improve nucleations but increasing filler content restricts crystal growth^37^. Figure 5b shows the exothermic crystallisation for different filler types. In the case of 50 wt% filler content, the crystallisation starts (T_c-onset_) at a significantly higher temperature due to the addition of different filler types than in pure PA6 sample (190.3 °C), clearly indicating an excellent heterogeneous nucleation effect of these filler types on the crystallisation of PA6 matrix. In the case of 50 wt% filler content, Al_2_O_3_ has the highest nucleation capacity since its T_c-onset_ is 195.1 °C, whereas the pure PA6 is 190.3 °C. Zeolite also has a high nucleation capacity, while Fritt1, Fritt2, and MgO fillers have slightly less. The crystallisation peak temperature (T_c_) also increases due to the influence of 50 wt% Al_2_O_3_, Fritt1, Fritt2, MgO, and natural zeolite. Increasing the filler content from 50 to 70 (and 80 wt% in the case of zeolite), the start crystallisation temperature (T_c-onset_) and crystallisation temperature (T_c_) are reduced to a minimal extent. Crystallisation behaves differently under the influence of MgO-Zeo hybrid filler. When MgO–Zeo hybrid filler is added, both the onset temperature and the peak temperature of the crystallisation are nearly the same as for the pure PA6 sample. But it is worth mentioning that the crystallization process depends not only on nucleation and growth but also on viscosity.

**Table 4:** Parameters obtained from DSC heating and cooling scans.

Sample	T_c-onset_, °C	T_c_, °C	ΔH_c_, J/g	T_m_, °C	ΔH_m_, J/g	X_c,_ %
PA6	190.3	184.6	61.85	223.6	55.64	23.2
PA6/Al_2_O_3_-50	195.1	189.2	33.63	221.2	25.07	10.4
PA6/Al_2_O_3_-70	193.4	188.34	23.24	221.4	16.53	6.9
PA6/MgO-50	193.0	187.1	27.88	222.4	20.51	8.5
PA6/MgO-70	187.4	181.1	17.58	221.1	14.11	5.9
PA6/Fritt1-50	193.4	187.2	29.07	221.7	24.69	10.3
PA6/Fritt1-70	194.7	188.7	27.95	221.4	23.68	9.9
PA6/Fritt2-50	193.4	187.8	31.51	221.8	29.68	12.4
PA6/Fritt2-70	191.8	186.4	22.79	221.5	16.7	7.0
PA6/Zeo-50	194.5	186.8	30.07	221.2	23.83	9.9
PA6/Zeo-70	193.1	186.1	26.89	221.0	24.16	10.1
PA6/Zeo-80	186.6	179.2	9.77	213.4	8.87	3.7
50%(PA6/MgO70) + 50%(PA6Zeo80)	190.3	183.8	14.68	219.2	12.14	5.1

### Thermal conductivity of the composite

The thermal conductivity of all investigated fillers is higher than the PA6 matrix (Table 3). Al_2_O_3_ has the highest thermal conductivity (36 W/mK), and MgO has slightly less conductivity (30 W/mK). The thermal conductivity of Fritt1, Fritt2, and natural zeolite is thirty times lower than that of MgO. The thermal conductivity of zeolites usually varies from 0.6 W/m K to almost 4 W/m K depending on the mine where the zeolite originated. Schnell et al.^42^ found a thermal conductivity of 1.2 W/m K for zeolite with a sodalite type framework by non-equilibrium molecular dynamics (NEMD) simulation.

**Figure 6:**
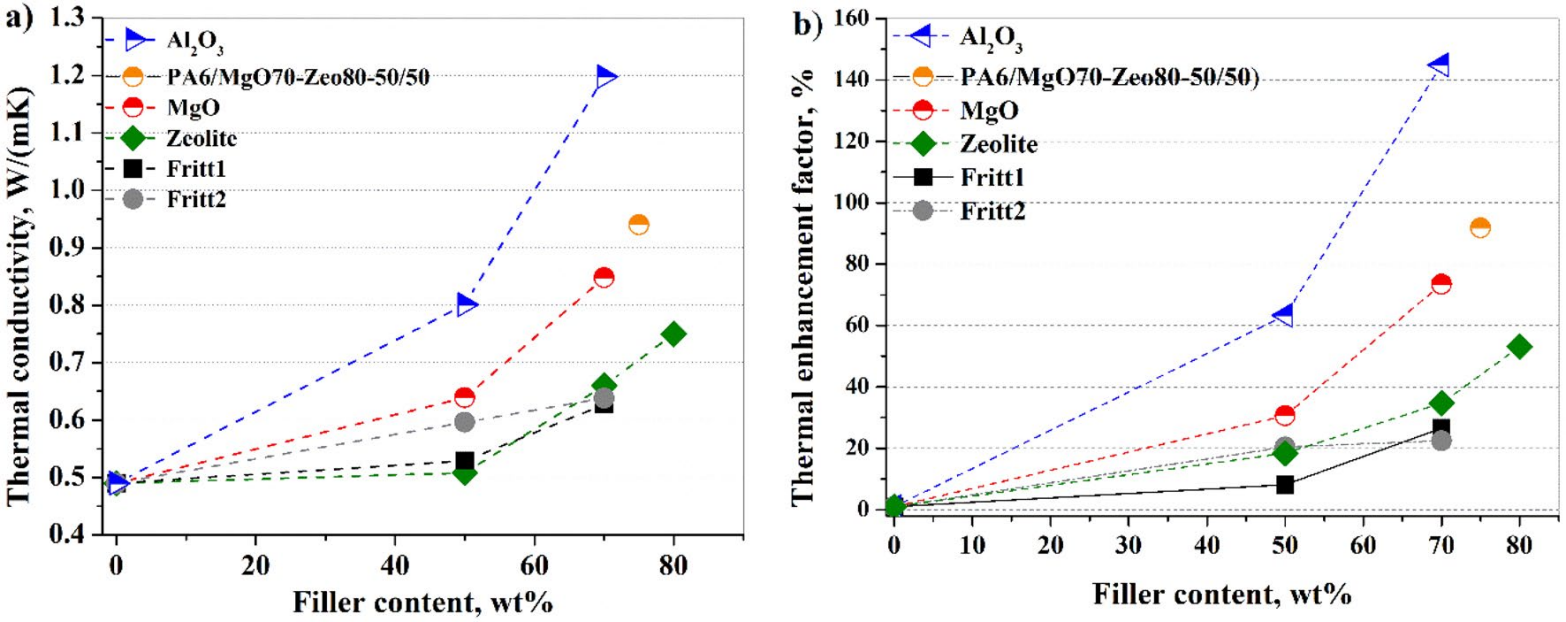
Thermal conductivity (**a**) of the composites with different filler types and weight fractions and thermal enhancement factor (**b**) of different types of filler.

The thermal conductivity of the samples as a function of the weight fraction of the fillers is shown in Fig. 6a and in Table 3. Every point of thermal conductivity is an average of three sample measurements. As can be seen, the conductivity of composites increases with the increase of the filler fraction. When the filler content is less than 50 wt%, the thermal conductivity increases slowly in the case of all the fillers. At 50% by weight, the percentage by volume of fillers varied between 24% (Al_2_O_3_) and 36% (zeolite). The heat-conductive particles generally surrounded by a polymer matrix cannot form a continuous path at low loading. The isolated particles do not significantly affect the enhancement of thermal conductivity. The heat transfer between the matrix and the filler occurs according to a serial model, and the thermal conductivity increases very slowly due to high thermal contact resistance. As the concentration of the filler increases, particles begin to touch each other to form a more compact packing structure. As the amount of filler increases, the distance between the filler particles decreases, eventually creating a network of filler contacts. So, thermal conductivity grows significantly because of reducing thermal contact resistance, and the tendencies are similar in Ref.^43,44^. Zijin Lin et al.^45^ proposed a factor (φ) that represents the variation in the thermal conductivity of the composites compared to the matrix. It was defined as follows:

3 $$\varphi =\frac{\lambda -{\lambda }_{p}}{{\lambda }_{p}}\cdot 100\%$$

where λ, and λ_p_ are composite and matrix thermal conductivity, respectively. As was expected, the highest thermal conductivity of Al_2_O_3_ increases the thermal conductivity of polyamide to the greatest extent. The thermal conductivity of the polymer does not increase to the expected extent in the case of MgO filler due to the uneven distribution of MgO particles. Fritt1, Fritt2, and zeolite have almost the same thermal conductivity value, but they demonstrate different thermal enhancement effects (Fig. 6b). As shown in Fig. 6b, natural zeolite is more effective in increasing the thermal conductivity of composite compared to Fritt1 and Fritt2. The average grain size of zeolite and MgO is approximately half that of Al_2_O_3_, Fritt1, and Fritt2. Thus, for a composite of equal mass and weight percent charge, taking into account the densities, significantly more particles are in the composite with natural zeolite or MgO than in the composites containing Al_2_O_3_, Fritt1, and Fritt2. The many zeolite particles increase the potential for the formation of heat conduction pathways. In addition, natural zeolite particles contain very thin layers, which can be easily displaced due to weak van der Waals bonding force, and thus nano-sized layers can be formed during mixing in the composite. Increasing the filler loading leads to more filler–filler interfaces. The interfacial thermal resistance (ITR) value for the PA6/ natural zeolite interface is probably smaller than the ITR for PA6/Fritt1 and PA6/Fritt2 interfaces.

The advantage of a hybrid filler system can be observed in Fig. 6. Two composites (containing PA/MgO-70/Zeo80-50/50) were mixed, and the thermal conductivity was 0.94 W/mK. In this mixture composite, the volume content of MgO is 27.46%, while zeolite is 34.69; namely, 62.15 V/V% is the total filler content. Separately the thermal conductivity of the MgO-containing composite is 0.85 W/mK, and that of the zeolite-containing composite is equal to 0.75 W/mK, while when mixed in 50–50 wt.%, the thermal conductivity reached 0.94 W/mK. The mixing of two composites with lower thermal conductivity resulted in higher thermal conductivity. This increase could only be achieved by creating more thermal bridges in the composite. Namely, the gaps between the zeolite particles were filled with MgO particles, and this was confirmed by microscopic examination. The value of the interfacial thermal resistance between zeolite and MgO particles is lower than that between PA6 and any of the fillers. The thermal conductivity of the polymer composite is doubled by using a MgO-zeolite hybrid filler. The price of natural zeolite is much lower than synthetic MgO, so the use of zeolite also has an economic advantage.

### Thermal conductivity models

There are numerous theoretical and empirical correlations in the literature for calculating the thermal conductivity of solid filler polymers. The simplest models of two-phase systems are the Parallel and Series models^46^, which are often used to predict the thermal conductivity of a composite. These models assume that the blocks of the polymer are arranged in series or parallel to the direction of thermal flux. The thermal conductivity of composite in Parallel and Series conditions can be estimated respectively by the following equation:

Series model

4 $$\lambda =\frac{{\lambda }_{p}\cdot {\lambda }_{f}}{{\lambda }_{f}\cdot \left(1-{V}_{f}\right)+{\lambda }_{p}{V}_{f}}$$

Parallel model

5 $$\lambda =\left(1-{V}_{f}\right)\cdot {\lambda }_{p}+{V}_{f}\cdot {\lambda }_{f}$$

where λ, λ_p_, and λ_f_ are the thermal conductivity of the composite, polymer, and filler, respectively; V_f_ is the volume fraction of the filler.

The Maxwell equation takes into account the volume fraction and thermal conductivity of filler and polymer with the assumptions of dispersion of small particles within a continuous matrix phase and the filler particles being far from each other^47^:

6 $$\frac{\lambda }{{\lambda }_{p}}=1+\frac{3\cdot (-1+\frac{{\lambda }_{f}}{{\lambda }_{p}})\cdot {V}_{f}}{\left(\frac{{\lambda }_{f}}{{\lambda }_{p}}+2\right)-{V}_{f}(\frac{{\lambda }_{f}}{{\lambda }_{p}}-1)}$$

This formula was valid only in the case of low filler volume (under 25%).

The Maxwell-Eucken model^48^ assumes that the uniform filler particles with no interaction are randomly distributed in the polymer matrix:

7 $$\lambda ={\lambda }_{p}\cdot \left[\frac{2\cdot {\lambda }_{p}+{\lambda }_{f}+2{V}_{f}\cdot ({\lambda }_{f}-{\lambda }_{p})}{2\cdot {\lambda }_{p}+{\lambda }_{f}-{V}_{f}\cdot ({\lambda }_{f}-{\lambda }_{p})}\right]$$

Lewis and Nielsen’s model^49^ takes into account the shape and orientation of the filler:

8 $$\lambda ={\lambda }_{p}\cdot \frac{1+AB{V}_{f}}{1-B{V}_{f}\Psi }$$

Here B $=\frac{\frac{{\lambda }_{f}}{{\lambda }_{p}}-1}{\frac{{\lambda }_{f}}{{\lambda }_{p}}-A}$, $\Psi =1+{V}_{f}\cdot \frac{1-{\phi}_{m}}{{{(\phi}_{m})}^{2}}$. The values of A and ɸ_m_ for many geometric shapes and orientations are given. (In our calculation A = 1.5 -spherical shape, ɸ_m_ = 0.82-maximum packing fractions for uniaxial random arrangements.)

The Effective Medium Theory (EMT) model is an implicit form and it considers other forms for spherical and ellipsoidal inclusions for conductivity^50^. It tackles materials with a completely random distribution of all the particles.

9 $$\left(1-{V}_{f}\right)\cdot \frac{{\lambda }_{p}-\lambda }{{\lambda }_{p}+2\lambda }+ {V}_{f}\cdot \frac{{\lambda }_{f}-\lambda }{{\lambda }_{f}+2\lambda }=0$$

The Reciprocity model^51^ assumes that a microstructure of two components remains statistically equivalent when exchanging the volume fractions of the components:

10 $$\frac{\lambda }{{\lambda }_{p}}=\frac{1+(\sqrt{\alpha }-1)\cdot {V}_{f}}{1+(\sqrt{1/\alpha }-1)\cdot {V}_{f}}$$

where $$\mathrm{\alpha }={\lambda }_{f}/{\lambda }_{p}$$.

The Bruggeman model^52^ generally is applicable for higher filler content. This is the implicit form:

11 $$1-{V}_{f}=\frac{{\lambda }_{f}-\lambda }{{\lambda }_{f}-{\lambda }_{p}}\cdot {\left(\frac{{\lambda }_{p}}{\lambda }\right)}^{1/3}$$

**Figure 7:**
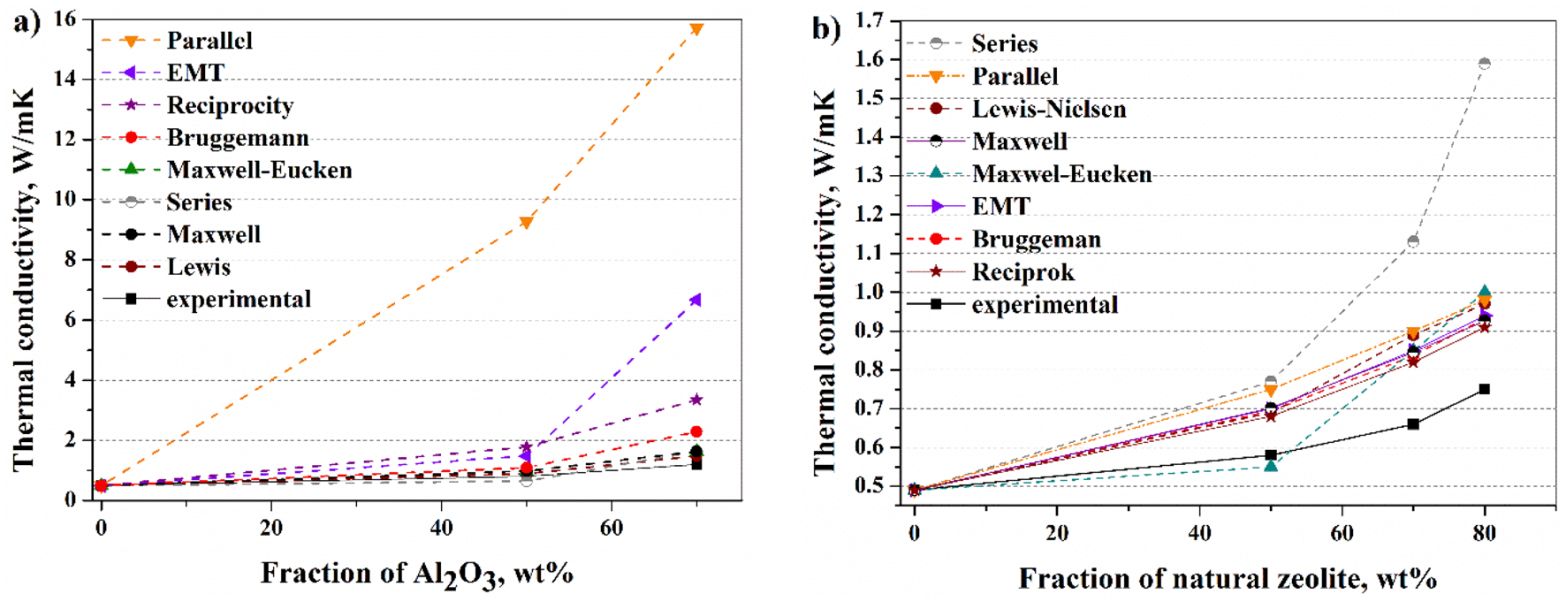
Comparisons between predicted and measured thermal conductivities of Al_2_O_3_ (**a**) and natural zeolite (**b**) containing composites.

The experimental data are compared with the results predicted by theoretical models. Table 5 lists the values of the experimental data and the thermal conductivities calculated by different models. In our experiment, the smallest volume filler content is 24%. The most significant difference between the calculated and experimental values is found for high thermal conductivity Al_2_O_3_ (Fig. 7a) and MgO filler in the parallel model. One reason for this is probably that the volume fraction of Al_2_O_3_ filler was the smallest and no proper heat dissipation pathway was formed. In the case of MgO, the filler distribution was not uniform. Lewis–Nielsen’s model gives the best approximation for Al_2_O_3_, Fritt1, Fritt2, and natural zeolite. The maximum deviation for these materials was 26%. Good agreement was found by the Reciprocity model for Fritt1, Fritt2, and natural zeolite. The maximum error is 25%. Observing the structure of the composites (Fig. 3b,d), the distribution of these fillers in the composites is indeed the closest to the Reciprocity model. Despite the simplicity of the Series model, its predictions give the best approximation of the experimental results for MgO filler content composite (Fig. 3c, Table 5). In the case of zeolite filler (Fig. 7 b), up to 50 wt%, the error of the Maxwell–Eucken model is less than 5%, but above 50% weight fraction, it is already more than 30% higher than the measured value. At our concentrations, the values calculated by the other models are much higher than the experimental values except in two cases (Series model-Al_2_O_3_-50 wt%, Maxwell–Eucken model natural zeolite 50 wt%). There may be several reasons for this discrepancy. The higher calculated thermal conductivity may be attributed to the fact, that these models cannot consider the high interfacial thermal resistance and the thin gap between the filler and the polymer matrix. Furthermore, if the filler distribution is not uniform, a significant deviation between the theoretical value and the measured value is possible.

**Table 5:** Comparisons between experimental results and existing theoretical solutions.

Filler	wt.%	V/V%	λ, W/mK								
			Exp	calculated λ, W/mK							
				1	2	3	4	5	6	7	8
MgO	50	34.21	**0.64**	0.74	10.59	1.20	1.28	1.28	2.33	3.05	1.56
	70	54.75	**0.85**	1.07	16.6	2.09	2	1.98	4.44	10.43	3.78
Al_2_O_3_	50	24.43	**0.8**	0.64	9.27	0.98	0.98	0.88	1.78	1.48	1.09
	70	42.92	**1.2**	0.85	15.73	1.63	1.63	1.41	3.35	6.67	2.29
Fritt1	50	29.92	**0.53**	0.69	0.71	0.66	0.67	0.65	0.65	0.66	0.66
	70	49.83	**0.62**	0.97	0.86	0.81	0.81	0.79	0.78	0.81	0.80
Fritt2	50	30.81	**0.59**	0.70	0.71	0.66	0.67	0.65	0.65	0.66	0.66
	70	50.88	**0.64**	0.99	0.86	0.80	0.80	0.78	0.78	0.80	0.80
Zeolite	50	36.51	**0.58**	0.77	0.75	0.69	0.55	0.68	0.68	0.70	0.69
	70	57.22	**0.66**	1.13	0.90	0.83	0.85	0.83	0.82	0.85	0.84
	80	69.62	**0.75**	1.59	0.98	0.92	1.00	0.94	0.91	0.94	0.93

1: Series model, 2: Parallel model, 3: Maxwell model, 4: Maxwell-Eucken model, 5: Lewis-Nielsen’s model, 6: Reciprocity model, 7: EMT model, 8: Bruggemann model.

Significant values are in [bold].

There are several models for hybrid filler. In the additive approach by Spencer et al.^53^, the individual contributions of the two fillers are summed without double counting the contribution of the matrix:

12 $$\frac{{\lambda }_{add}}{{\lambda }_{p}}=\frac{{\lambda }_{f1}}{{\lambda }_{p}}+\frac{{\lambda }_{f2}}{{\lambda }_{p}}-1$$

where λ_add_ is the thermal conductivity of hybrid filler composite using two types of filler. λ_f1_ and λ_f2_ are the composite thermal conductivity, with f_1_ and f_2_ containing fillers, respectively.

Woodside–Messmer^54^ proposed a quadratic parallel model for a two-phase hybrid composite:

13 $$\lambda =\sqrt{{\lambda }_{p}}\cdot {V}_{p}+\sqrt{{\lambda }_{f1}}\cdot {V}_{f1}+\sqrt{{\lambda }_{f2}}\cdot {V}_{f2}$$

where λ_f1_ and λ_f2_ are the thermal conductivity of f_1_ and f_2_ fillers, respectively. V_p_, V_f1_ and V_f2_ are the volume fraction of the matrix, f_1_, and f_2_ fillers.

Using these two models for MgO-zeolite hybrid filler composite, the thermal conductivity is 1.11 and 2.15 W/mK according to the additive and quadratic parallel model. The measured thermal conductivity of the hybrid filler is 0.94 W/mK. The additive approach proves to be a good approximation.

### Abrasion wear test

**Figure 8:**
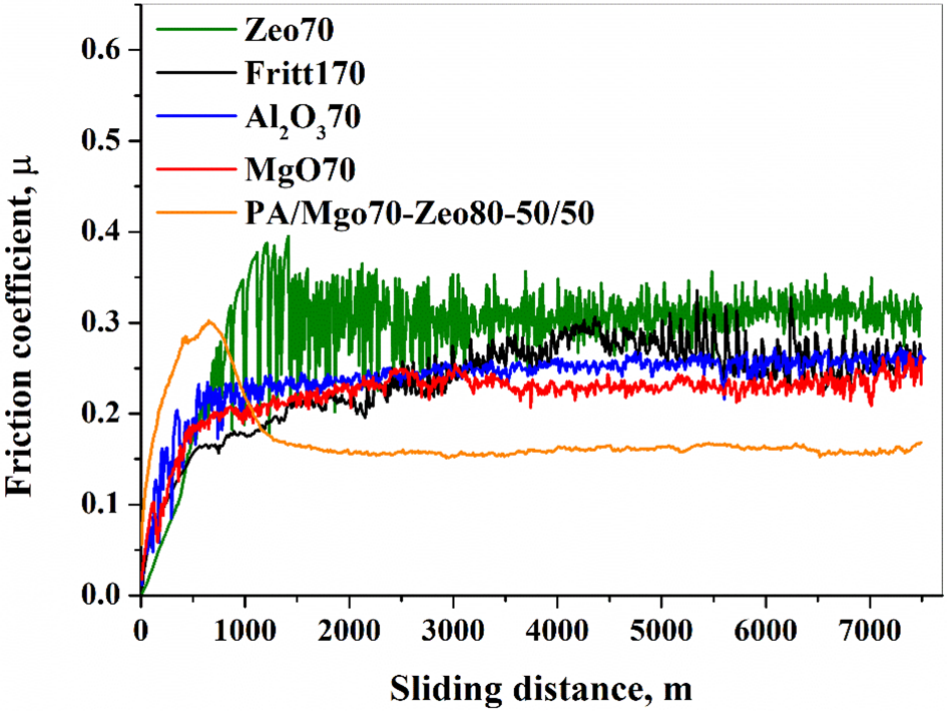
Friction coefficient curves with different types of fillers and filler content.

**Figure 9:**
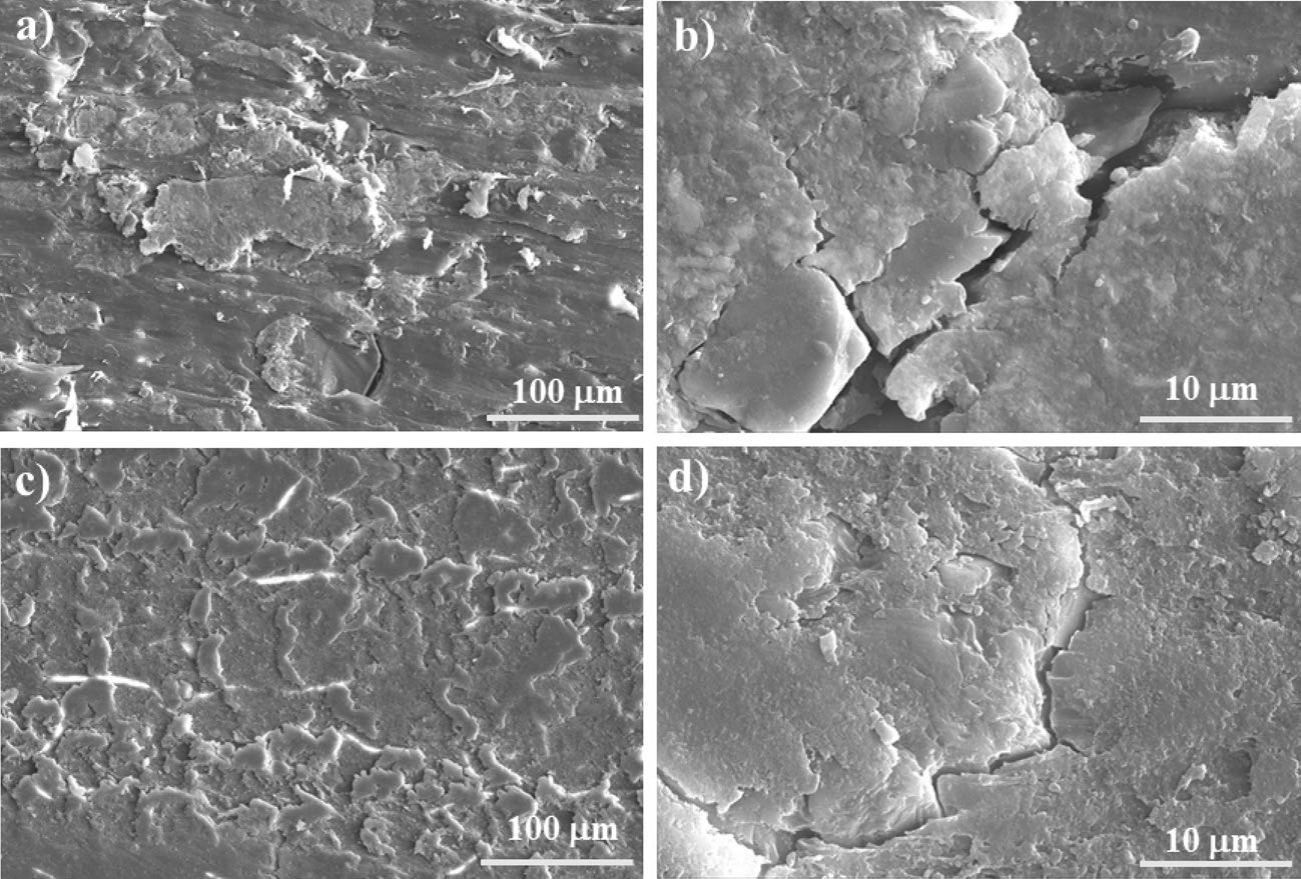
Scanning electron microscope images of wear tracks after 7000 m of sliding: (**a**), (**b**) PA6/Al_2_O_3_-70, (**c**), (**d**) PA6/Zeo-70.

Figure 8 represents the friction coefficient traces recorded during the pin-on-disc tests of five differentPA6-based composite specimens. Similar observations were also recorded for other samples. During the initial wear stage, the friction coefficient curves increase rapidly. After a certain distance, a steady state friction coefficient was obtained after a run-in of about 2000 m. However, it is apparent that the natural zeolite-filled composite exhibits pronounced fluctuations in friction coefficient throughout the test until 2000 m is reached. Between 2000 and 7500 m sliding distance, the zeolite-filled composite has the highest friction coefficient value (0.31 ± 0.02) and the hybrid-filled composite has the lowest friction coefficient (µ:0.16 ± 0.004). The friction coefficient of MgO, Fritt1, and Al_2_O_3_ filled composites are 0.23 ± 0.01, 0.25 ± 0.01, and 0.26 ± 0.02, respectively. Observing the worn surface of the Al_2_O_3_-70 filled composite (Fig. 9a) it can be stated that debris are present on the worn surface, which supposes the formation of adhesive wear. The higher magnification SEM image (Fig. 9b) clearly shows that the Al_2_O_3_ grain and the PA6 matrix have separated due to the tensile stress. The edges of the Al_2_O_3_ grains on the surface produce a strong cutting effect. In the case of natural zeolite-filled composite, the particles of average particle diameter are partially or completely covered by the polymer matrix. Thin flakes have separated from the surface. Definite cutting edges are not visible during abrasion (Fig. 9c), the filler particles are not broken, but cracks are created between the matrix and the grains due to tensile stress, and then fall out of the matrix (Fig. 9d).

**Figure 10:**
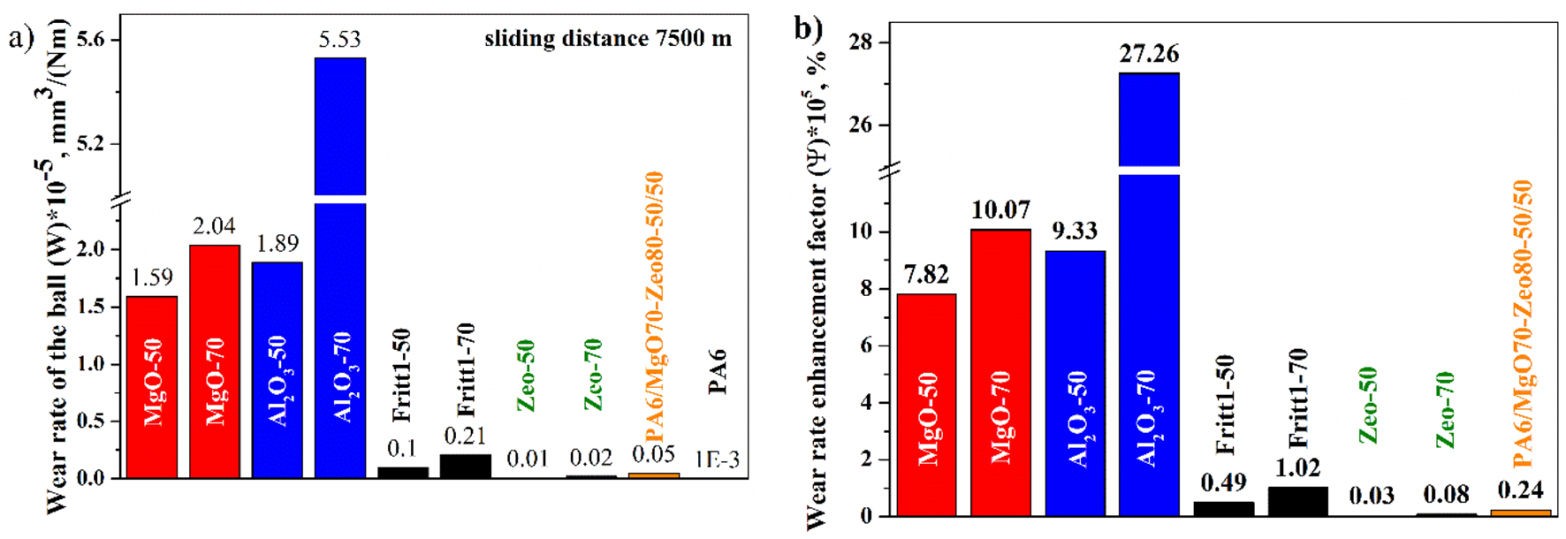
Specific wear rate with different types of fillers and filler content (**a**) and specific wear rate enhancement factor (**b**).

W_r_ is the tool wear during the composite production estimated by the volume loss of the steel ball. The ball's wear rate characterises the composite's abrasive effect in our case. A smaller wear rate of the steel ball means a smaller abrasive effect on the composition. Figure 10a depicts the variation of specific wear rates with an increase in different filler weight percentages in PA6. As the reinforcement of filler is increased, a general development trend in the wear rate of the ball on the composite materials is observed. Comparing the abrasive effects of all five types of composites, it is found that the abrasive rate value for natural zeolite reinforced composite is the lowest, followed by Fritt1, Fritt2, MgO, and Al_2_O_3_. There is no linear relationship between the abrasive effect and the hardness of the composite, because the hardest composite was the 70 wt% natural zeolite composite. In comparison, the least hard composite was the 50 wt% Al_2_O_3_ composite. However, if you look at the hardness of the fillers, the Al_2_O_3_ filler has the highest hardness, followed by MgO, and then nearly equal hardness for Fritt1, Fritt2, and zeolite (Table 3). Mohs hardness of a steel is about 4. So it is understandable that Al_2_O_3_ and MgO grains, when they are brought to the surface during abrasion, will greatly increase the wear of the softer steel tool. Similar to the thermal enhancement factor proposed by Lin et al.^45^, we have introduced an *abrasive effect factor* = wear rate enhancement factor that shows by what percentage the abrasive effect of the polymer has increased as an effect of the filler content:

14 $$\uppsi = \frac{{W}_{r,c}-{W}_{r,m}}{{W}_{r, m}} 100 (\mathrm{\%})$$

In the above equation W_r,c_ and W_r,m_ are the specific wear rate of composite and polymer matrix, respectively. The abrasive effects of the composites with Al_2_O_3_ and MgO filler were more than 100 times higher than that of PA6 with hybrid filler or natural zeolite filler (Fig. 10b). The advantage of the hybrid filler is also reflected in the abrasive effect, as the PA/MgO-70/Zeo80-50/50composite contains 35 wt% MgO and 40 wt% natural zeolites, the strong abrasive effect of MgO is significantly reduced.

**Figure 11:**
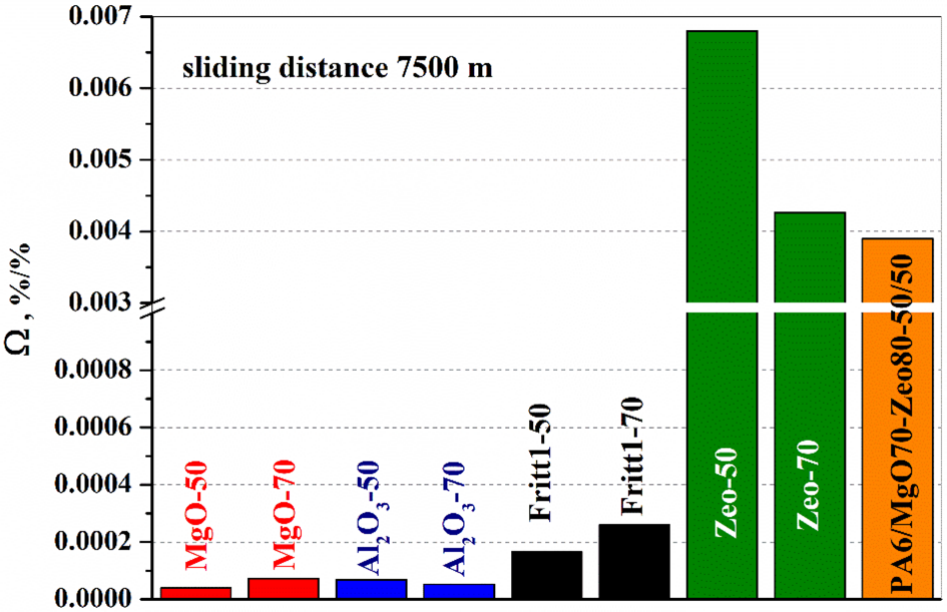
Rate of thermal and wear rate enhancement factor.

In the case of polymer composites with good thermal conductivity but electrical insulation, one should also take into account the tool wear caused by ceramic fillers during production. MgO and Al_2_O_3_ fillers, which increase thermal conductivity the most, also cause significant tool abrasion. The optimal filler material should increase thermal conductivity while causing less tool wear. Taking this into consideration, a process *efficiency factor* is defined as:

15 $$\Omega =\frac{\varphi }{\psi } , \frac{\%}{\%}$$

where φ and ψ are thermal and wear rate enhancement factors, respectively. A high value of for Ω factor means that the filler not only increases heat conduction, but also does not cause too much tool wear. As shown in Fig. 11, natural zeolite is more effective in increasing the thermal conductivity of the composite with less tool wear compared to the other filler contents. The use of hybrid filler is also preferable to Al_2_O_3_, Fritt1-2, or MgO.

## Conclusions

The thermally conductive and electrical insulating PA6 composites contain Al_2_O_3_, MgO, Fritt1, Fritt2 glasses, natural zeolite particles, and MgO-natural zeolite hybrid as fillers were successfully fabricated by injection moulding. The following conclusions are drawn:

- Each filler modified the crystallisation and melting of PA6.
- The coefficient of thermal conductivity varied from 0.53 to 1.2 depending on the quality and quantity of the filler, which is increasing by from 8 to 144% in comparison to the pure PA6.
- The thermal conductivity enhancement factor is highest for Al_2_O_3_ (145%), and MgO-zeolite hybrid fillers (92%).
- The Lewis–Nielsen and Reciprocity models gave the best approximations to the experimental results. The difference was less than 26%, except for the MgO-loaded composites.
- A new process efficiency factor (Ω) was developed that takes into account the manufacturability. A high value of the Ω factor means: not only does the filler increase heat conduction, but it also does not cause too much tool wear.
- Considering tool wear caused by fillers, natural zeolite and MgO-natural zeolite hybrid filler are much preferable compared to Al_2_O_3_, Fritt1-2 glass, or MgO. Among heat-conducting composites, the use of hydrosilicate raw materials, which can be mined in large quantities, can reduce the energy demand for the production of the composite.

## Data Availability

The datasets used and analysed during the current study available from the corresponding author on reasonable request.
